# Competition between strain and dimensionality effects on the electronic phase transitions in NdNiO_**3**_ films

**DOI:** 10.1038/srep18707

**Published:** 2015-12-21

**Authors:** Le Wang, Sheng Ju, Lu You, Yajun Qi, Yu-wei Guo, Peng Ren, Yang Zhou, Junling Wang

**Affiliations:** 1 School of Materials Science and Engineering, Nanyang Technological University, Singapore 639798, Singapore; 2 School of Physical Science and Technology, Soochow University, Suzhou 215006, China; 3 School of Materials Science and Engineering, Hubei University, Wuhan 430062, China

## Abstract

Transition metal oxides host an array of exotic electronic phases, including superconductivity, ferroelectricity, quantum spin liquid and Mott insulators. Their extreme sensitivity to external stimuli enables various routes to manipulate the ground state, which greatly improves our understanding of the physics involved. Here, we report the competition between strain and dimensionality effects on the phase evolution in high quality NdNiO_3_ films down to several unit cells. While both compressive and tensile strains increase the Ni *3d* band width and favor the metallic phase, reducing dimensionality, on the other hand, decreases the covalent band width and favors the insulating phase in NdNiO_3_. The experimental observations are well supported by *ab initio* calculations and improve our understanding of the electronic behavior in strongly correlated electron systems.

Transition metal oxides are fascinating materials in which the interplay between charge, spin, orbital and lattice degrees of freedom leads to many exotic phenomena^1^,^2^. Understanding their unusual electronic behavior has been a long-standing task. The challenge is to treat simultaneously the strong correlation among the transition-metal *3d* electrons and their hybridization with oxygen (O) *2p* electrons. If the hybridization can be neglected, the physics would be represented by the Hubbard model involving the *3d* electrons only. However, in many cases the *2p*-*3d* charge transfer is large enough that the *2p* orbitals cannot be ignored^3^. Consequently, strain engineering has been widely adopted to tune this charge transfer energy and the electronic properties of transition metal oxides^1^,^4^,^5^,^6^. At the same time, it is well known that reducing dimensionality affects the electron correlation effect^7^,^8^,^9^,^10^. It is thus interesting to study how these two factors interact and compete in one system.

Bulk rare-earth nickelates, RNiO_3_ (R≠La), which undergo a first order metal-insulator transition (MIT) at temperature *T*_*MI*_ and a paramagnetic-antiferromagnetic transition at the Neel temperature (*T*_*N*_), have recently attracted much interest^11^,^12^,^13^,^14^,^15^,^16^. The high-temperature metallic phase is believed to be formed due to Δ<*W*^17^, where Δ and W are the charge-transfer energy and the O *2p*-Ni *3d* hybridization strength or covalence bandwidth, respectively. However, the nature of the low-temperature insulating phase is still under debate^18^,^19^,^20^. Recently, it was proposed that partial charge disproportionation (CD) between the Ni sites accompanied by a symmetry change from orthorhombic to monoclinic lowers the potential energy and gaps the Fermi surface^21^,^22^,^23^,^24^. Because the O *2p* levels lie very close to the initially empty nickel (Ni) *3d* levels, some of the O *2p* electrons jump to Ni *3d* levels and leave holes in the O *2p* band, so that RNiO_3_ have been classified as small or negative charge transfer systems^25^. The small charge transfer energy and strong correlation among 3d electrons in RNiO_3_ makes them very sensitive to external perturbations, providing an ideal playground for investigating the multiple degenerate ground states.

Advances in thin film growth technologies make it possible to obtain ultra-thin RNiO_3_ films and open the window for investigating dimensionality effect and its competition with other factors in these materials. Here, we have studied a set of NdNiO_3_ (NNO) films with different thicknesses in an attempt to figure out how strain and dimensionality together affect the electronic phase evolution. By varying NNO film thickness from 400 unit cells (u.c.) to 5 u.c., we uncover the critical thickness (~20 u.c.) in this system, above which strain effect dominates the phase evolution by increasing the Ni 3d band width and favors the metallic phase (decrease *T*_*MI*_). Below the critical thickness, dimensionality effect begins to dominate the phase change by decreasing the covalent band width and favors the insulator phase (increase *T*_*MI*_). Our results reveal how the phase diagram of NNO can be manipulated by controlling the thickness of NNO films, which highlights the power of hetero-interface engineering and its potential for applications in nano-electronics.

## Results and Discussions

### Sample preparation and characterizations

**Figure 1:**
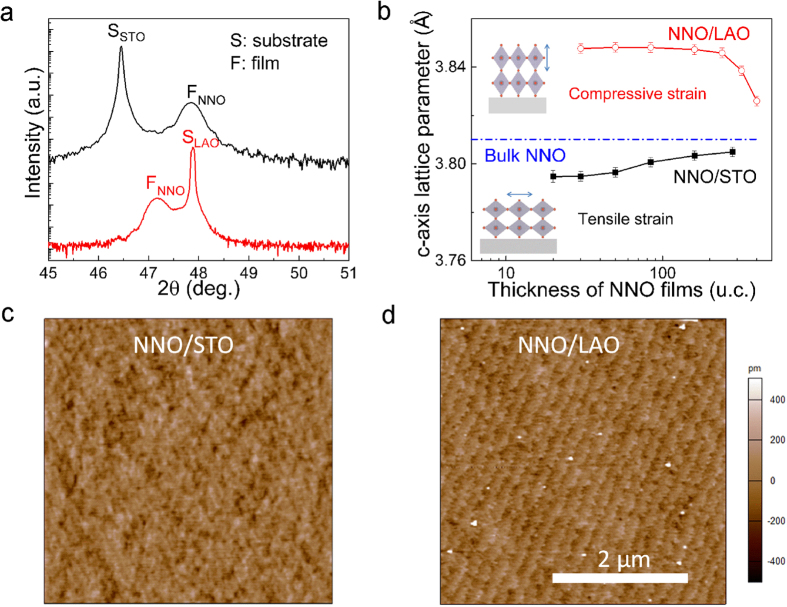
Sample preparation and structural characterization. (**a**) XRD *θ*−2*θ* scans around the (002) peaks of 84 u.c. NNO films on the two different substrates. The ‘S’ and ‘F’ denote the substrates and NNO films, respectively. Clear Kiessig fringes indicate the high quality of the films. (**b**) *c*-axis lattice parameters as functions of NNO film thickness. The insets show the lattice distortion under different strains, respectively. Topography images of 20 u.c. NNO films on (**c**) STO and (**d**) LAO substrates, respectively.

We have deposited high-quality epitaxial NNO thin films using pulsed laser deposition on (001)_c_-oriented SrTiO_3_ (STO) and (001)_pc_-oriented LaAlO_3_ (LAO) substrates (the subscripts ‘c’ and ‘pc’ represent cubic and pseudocubic structures, respectively). In bulk, NNO is orthorhombic with room temperature pseudocubic lattice constant of ~3.81 Å, which is larger than that of LAO (a_LAO_ ~ 3.794 Å), but smaller than that of STO (a_STO_ ~ 3.905 Å). Therefore, NNO experiences a compressive strain of −0.4% on LAO and tensile strain of 2.5% on STO, respectively. Figure 1a shows the *θ* − 2*θ* scans around (002) peak of the 84 u.c. NNO films grown on these two substrates. Films with thicknesses below 30 u.c. are found to be coherently strained on both substrates (Fig. 1b), consistent with previous work^26^. When the film thickness is below 20 u.c., the diffraction peak broadens (Supplementary Fig. S1), leading to increased error bar in the calculated out-of-plane lattice constant. The topography images reveal atomically flat surfaces with a mean roughness of 0.2 nm for both films (Fig. 1c,d). The step flow growth assures high quality of the films.

**Figure 2:**
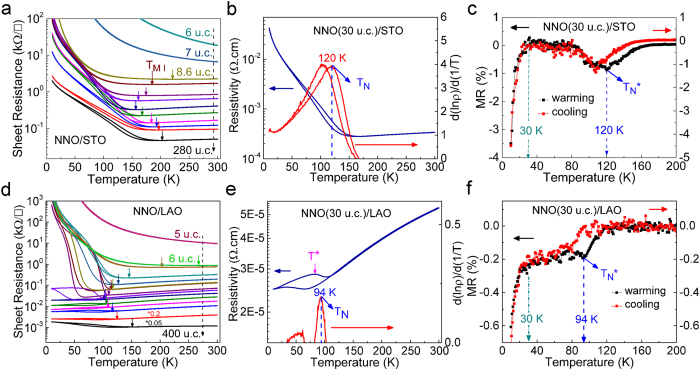
In-plane transport measurements. Sheet resistance (*R*_*sheet*_) versus temperature for NNO films with different thicknesses on (**a**) STO and (**d**) LAO substrates, respectively. Resistivity and 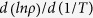 versus temperature for 30 u.c. NNO films grown on (**b**) STO and (**e**) LAO, respectively. *ρ* = *R*_sheet _* *d*, where *ρ* is the resistivity, and *d* is the thickness of NNO film. Temperature dependence of the MR, [R(H)-R(0)]/R(0), for 30 u.c. NNO films grown on (**c**) STO and (**f**) LAO, respectively. R(H) is the *R*_*sheet*_ under a magnetic field of H (10 T in this case), and R(0) is the zero-field *R*_*sheet*_. *T*_*MI*_, *T*_*N*_, and *T*_N_* are defined as the temperatures of the upturn in the R_sheet_ –T plot, the peak in the 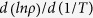 – T plot, and the peak in the MR - T plot on heating, respectively. T* denotes the temperature point, below which the metallic phase remains dominant.

Figure 2a shows the sheet resistance versus temperature relationships for NNO/STO films ranging from 280 u.c. to 6 u.c. As the thickness decreases, the overall sheet resistance increases continuously. However, two different regimes can be observed. *T*_*MI*_ decreases with reducing film thickness from 280 u.c. to 20 u.c., after which it quickly increases upon further reduction of the film thickness, accompanied by the destruction of the first-order nature of the MIT. Finally, for the 7 u.c. and thinner NNO films, insulating behavior is even observed at room temperature. Similar behavior is observed for films on LAO as shown in Fig. 2d (with a peculiar behavior for intermediate thickness, see discussion in Supplementary Fig. S2). Note that despite the similar thickness dependence, room temperature resistivity and *T*_*MI*_ of NNO films on LAO are lower than that on STO for the same thickness (Fig. 2b,e), which is consistent with previous reports^13^,^26^,^27^.

Apart from *T*_*MI*_, we can also obtain the magnetic phase transition temperature *T*_*N*_ following 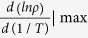 (see Fig. 2b,e), where ρ is resistivity on the insulating side. To further corroborate the values obtained, we have also carried out magnetoresistance (MR) measurements with the magnetic field perpendicular to the film surface. Negative MR effect (Supplementary Fig. S3) is observed and magnetic phase transition temperature, *T*_N_*, is extracted again (Fig. 2c,f). We notice that *T*_N_* is identical to *T*_*N*_. The MR value increases rapidly below 30 K, which has been attributed to the weak localization effect^28^.

### Phase diagrams of NNO/STO and NNO/LAO films

**Figure 3:**
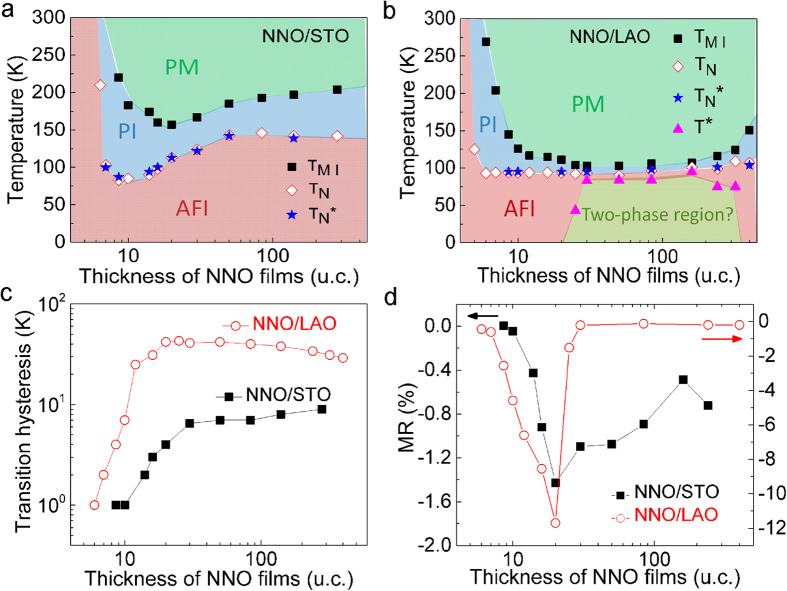
Phase evolution as a function of the film thickness. Phase diagrams for the (**a**) NNO/STO and (**b**) NNO/LAO systems, respectively. PM, PI, and AFI denote paramagnetic metal, paramagnetic insulator and antiferromagnetic insulator, respectively. Two-phase region denotes the region where the metallic and insulating phases coexist, and the temperature dependency of transport behavior is dominated by the metallic phase (see Supplementary Information for details). (**c**) R-T hysteresis width as a function of the NNO film thickness. Hysteresis width refers to the difference between *T*_*MI*_ upon heating and cooling. (**d**) Negative MR at *T*_*N*_ as a function of the NNO film thickness.

Using the transition temperatures (*T*_*MI*_, *T*_*N*_, *T*_N_*) obtained from transport measurements, we have constructed the phase diagrams of NNO films on both STO and LAO substrates (Fig. 3a,b). As temperature decreases, both films undergo the transitions from a paramagnetic metallic (PM) phase, through a paramagnetic insulating (PI) phase, to an antiferromagnetic insulating (AFI) phase. These three phases have all been found in bulk RNiO_3_ with smaller R (Lu through Sm) but not in bulk NNO, which defaults the PI phase^11^. The emergence of the PI phase implies the opening of a gap that is decoupled from the spin ordering. Moreover, two-phase region was also observed when the thickness of NNO films is among 25 u.c. and 320 u.c. in NNO/LAO (see Supplementary Information for details).

Looking at the thickness dependence, the phase diagrams can clearly be separated into two regions below and above a critical thickness of ~20 u.c., regardless of what is the substrate used. Above the critical thickness, *T*_*MI*_ and *T*_*N*_ decrease with thickness for both films. Below the critical thickness, they both increases quickly. Comparing the phase diagrams with the lattice evolution shown in Fig. 1b, it is likely that strain effect dominates in the thicker films, while other effect (e.g. dimensionality) takes over in the ultra-thin films (where strain doesn’t change anymore). The existence of the critical thickness is also supported by other analysis as shown in Fig. 3c,d. For example, the hysteresis width in the R-T curves starts to decrease quickly below 20 u.c. and maximum MR is observed for both films at around 20 u.c., regardless of the type of strain imposed by the substrate.

**Figure 4:**
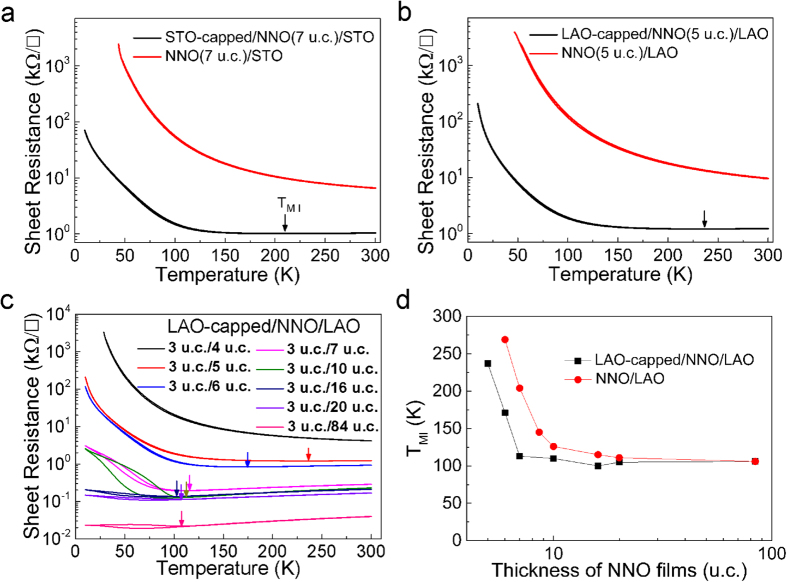
Transport properties of uncapped and capped ultrathin NNO films. (**a**) R_sheet_ versus temperature behavior of 7 u.c. NNO film on STO capped with 3 u.c. STO layer and uncapped. (**b**) R_sheet_ versus temperature behavior of 5 u.c. NNO film on LAO capped with 3 u.c. LAO layer and uncapped. The arrows show the *T*_*MI*_ points on heating. (**c**) R_sheet_ versus temperature for NNO films with different thicknesses capped with 3 u.c. LAO layer. (**d**) NNO thickness dependence of *T*_*MI*_ for NNO/LAO and LAO-capped/NNO/LAO structures.

Similar behavior has been reported in SrVO_3_, LaNiO_3_ and PrNiO_3_ ultra-thin films. Kumah *et al.* proposed that surface polar distortion, coupled with octahedral rotations, induced a systematic decrease in the average in-plane Ni-O-Ni bond angles, decreased W and increased *T*_*MI*_^29^. Others suggested that dimensional crossover could be the cause^30^,^31^,^32^. To check if surface effect plays a role in our system, we cap the ultra-thin NNO films with an STO (or LAO) insulating layer. As shown in Fig. 4a,b, capping the ultrathin NNO films with one insulator layer of STO or LAO can recover the room temperature metal behavior. Compared with the results of NNO/LAO structures shown in Fig. 2c, the LAO-capped/NNO/LAO structures show similar trend in the change of *T*_*MI*_ upon reducing film thickness (Fig. 4c,d), albeit at a slightly reduced critical thickness. Therefore, dimensional crossover should be the main driving force that stabilizes the insulating phase in ultra-thin nickelates films. This speculation is also consistent with the observed phenomena that compressive strain can only cause the appearance of unusual two-phase region under certain thickness range (between 25 u.c. and 320 u.c.). Once the film thickness is lower than the critical thickness (20 u.c.), the dimensionality effect begins to dominate the system state, and the two-phase region disappears from the phase diagram (shown in Fig. 3b).

### First principle calculations and effects of strain and dimensionality

To understand the experimental observations and support our analysis, we turn to *ab initio* calculations (see Methods for details). The idea is to obtain changes in the Ni-O-Ni bond and electronic structure in NNO under various conditions, from which we can understand the transport behavior. For bulk NNO, we start from experimentally identified crystal structure with space group *P21/n*. To simulate the epitaxial strain effect, the in-plane lattice constants of NNO are fixed to that of the STO or LAO substrate (3.905 Å and 3.794 Å), respectively, while the out-of-plane lattice is allowed to relax. On the other hand, to study the dimensionality effect, we use a NdAlO_3_(NAO)/NNO/NAO heterostructures, where two u.c. of NNO is sandwiched between neighboring wide band gap insulator NAO with no polar discontinuity at the interfaces.

**Figure 5:**
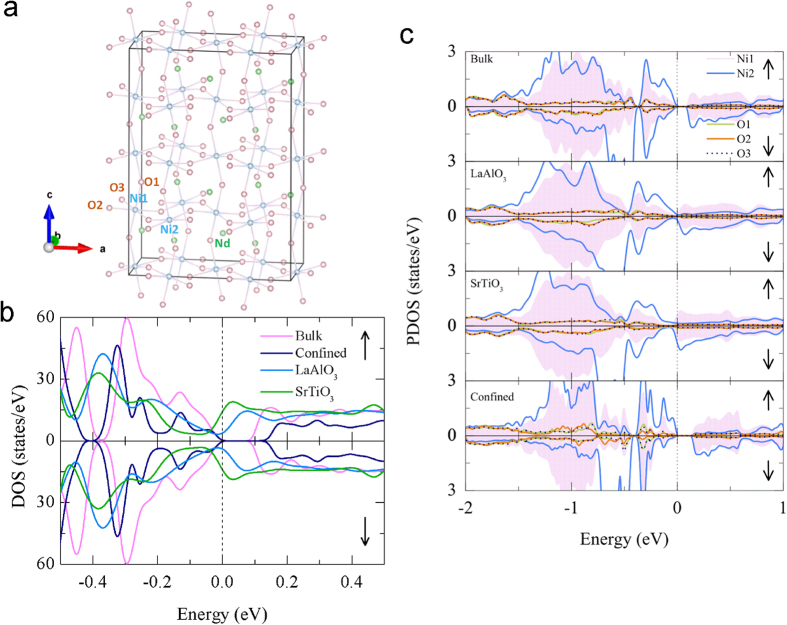
*ab initio* calculations. (**a**) The NNO supercell used for bulk calculations. (**b**) Density of states (DOS) of bulk NNO, epitaxially strained thin films on STO and LAO substrates, and confined system. (**c**) Partial DOS (PDOS) of the four different systems. The Fermi level is indicated by the dashed line.

The supercell used for the bulk calculations is shown in Fig. 5a with the O and Ni locations indicated. The ground state total density of states (DOS) of bulk NNO, epitaxially strained thin films on STO and LAO substrates, and confined system are shown in Fig. 5b. Without introducing the Coulomb correlation term (U), a small gap is revealed in bulk NNO with T-type AFM ordering, consistent with the insulating ground state. For the epitaxially strained thin films, both compressive and tensile strain reduce the band gap to zero, in qualitative agreement with the experimental observation that *T*_*MI*_ decreases upon reducing thickness in both cases (metallic phase becoming more stable). On the other hand, when the thickness of NNO is reduced to 2 u.c. as in the confined system, our calculation shows that the band gap is increased as compared with bulk. This indicates that insulating phase is stabilized in ultra-thin films, again consistent with our experimental observations.

The partial DOS (PDOS) shown in Fig. 5c reveals more about the effects of strain and dimensionality. The charge ordered state of NNO is evident by the two different Ni sites. For bulk NNO, Ni1 is almost nonmagnetized while Ni2 is spin-polarized. The band gap is opened between the occupied Ni2 t_2g_ states and unoccupied Ni2 e_g_ states, as well as e_g_ states of both spin channels at Ni1 sites. With epitaxial strain applied, either compressive or tensile, the 3d orbitals become more delocalized, disrupting the charge ordered band gap therein and stabilizing metallic phase. On the other hand, when the thin film is confined along the c direction, the Van-Hove singularity is introduced with dips observed in the DOS of Ni ions and the band gap is increased. In addition, the Ni1 sites are also spin-polarized to some extent, indicating that the confinement effect has also changed the magnetic property of the system.

Our results are in qualitative agreement with that of the recent studies by angle-resolved photoemission spectroscopy (ARPES)^33^,^34^,^35^. Both tensile and compressive strain change the Fermi surface pockets and thereby control the Fermi surface nesting, and decrease *T*_*MI*_. The compressive strain reduces the crystal field splitting in NNO/LAO, lowering the e_g_ states and lifting those of the t_2g_ orbitals. This leads to the appearance of a new holelike Fermi surface at the corner of the Brillouin zone^33^, which plays a crucial role in obtaining a lower *T*_*MI*_. On the other hand, dimensional crossover could induce the orbital reconstruction and Fermi Surface nesting effect^10^,^35^, which result in the insulating state for the 2D ultrathin films. Moreover, the disorder effects, such as Anderson localization^35^, could also be important to understand the insulating state of the 2D ultrathin NNO films.

To summarize, we have revealed how strain and dimensionality affect the electronic structure of NNO films and conclude that a critical thickness of ~20 u.c. is identified, above which the net effect of strain (whatever tensile or compressive) reduces *T*_*MI*_ and favors the metallic phase, below which dimensionality decreases W and favors the insulating phase. The dimensional crossover changes NNO at room temperature from a low-∆ metal to a charge transfer insulator (W<∆<U) for the ultra-thin films. This is accompanied by the destruction of the first-order nature of the MIT. The experimental observations are well supported by *ab initio* calculations. Our work significantly improves our understanding of the electronic behavior of nickelates, and should be of great interest to the large audience working on strongly correlated electron systems in general. Extending our results to other nickelates would also enable us to address long-standing disputes, such as the issue of whether the sign of the strain affects the sign of the shift in the electronic phase transition temperature, and to design the related electronic devices based on nickelates.

## Methods

### Sample preparation

The NNO films are deposited on (001)_c_-oriented STO and (001)_pc_-oriented LAO substrates using pulsed laser deposition. The laser pulse (248 nm) energy density is ~2 J/cm^2^ and the repetition rate is 5 Hz. During the deposition, the substrate is kept at 630 °C under an oxygen pressure of 40 Pa. After deposition, we raise the oxygen pressure to 10 kPa and cool the samples to room temperature. The surface morphology of the NNO films is examined using atomic force microscope (AFM).

### Structure characterization

X-ray diffraction and reflectivity measurements are performed using a Rigaku SmartLab instrument. STO (002) peak is used as the reference for sample alignment. Inter-planar spacing along the c-axis is determined following Bragg’s law: n⋅λ = 2⋅d⋅sinθ, where integer n denotes the reflection order, λ is the x-ray wavelength, which is 1.54055 Å for Cu Kα radiation, d is the inter-planar spacing and θ is the diffraction angle. The thickness of the films is determined using x-ray reflectivity data and transmission electron microscopy (TEM) results.

### Electrical transport measurements

In-plane transport measurements are conducted using a 14 tesla (T) PPMS (physical properties measurement system, Quantum Design) system at temperatures ranging from 10 to 300 K at a cooling/warming rate of 3 K/min. We use linear four point geometry with Pt top electrodes to make Ohmic contacts. The magnetoresistance of the NNO films are measured with a perpendicular magnetic field.

### *ab initio* calculations

Our *ab initio* calculations are performed using the accurate full potential projector augmented-wave (PAW) method^36^ as implemented in the Vienna ab initio Simulation Pack-age (VASP)^37^. The generalized gradient approximation (GGA) in the form proposed by Perdew, Burke, and Ernzerhof (PBE) is used^38^. A large plane-wave cutoff 500 eV is used for all calculations and the convergence criteria for the energy is 10^−5^ eV. The PAW potentials are used to describe the electron-ion interaction, with 11 valence electrons for Nd^3+^ ion with 5s^2^5p^6^ and 4f^ 3^ frozen in the core, 16 for Ni (3p^6^3d^8^4s^2^), and 6 for O (2s^2^2p^4^). In our calculations, ions are relaxed until the Hellman-Feynman forces are less than 1 meV/Å and the c axis is optimized. An 8 × 8 × 6 Monkhorst-Pack *k*-point mesh centered at the Γ point is used for the 20-atom unit cell. For larger supercells, *k*-point mesh is reduced accordingly. For the electronic density of states (DOS) calculations, a denser *k*-mesh of 16 × 16 × 12 is used for the Brillouin zone integrations. By comparing the total energies of bulk NNO with normal ferromagnetic (FM) ordering, G-type anti-ferromagnetic (AFM) ordering, C-type AFM ordering, A-type AFM ordering, E-type AFM ordering, E^*^-type AFM ordering, S-type AFM ordering, and T-type AFM ordering, T-type AFM ordering is the ground state (Supplementary Fig. S4).

The bulk NNO possesses a monoclinic crystal structure with space group *P21/n* and the Ni ions have two different atomic positions with oxygen octahedral elongated for one Ni and compressed for the other. For the T-type AFM ordering, a 2 × 1 × 2 supercell with 80 atoms is used. To simulate the strain effect solely, we use bulk-strain calculations, where the in-plane lattice constant is fixed to the experimental substrates while the out-of-plane lattice constant is optimized. On the other hand, to isolate the quantum confinement effect (dimensionality effect), 2 layers of NNO are sandwiched between two NAO layers in a 2 × 1 × 2 supercell and the crystal structures are optimized freely (Supplementary Fig. S5). The details about the structural parameters in the calculated process are shown in Supplementary Table S1.

## Additional Information

**How to cite this article**: Wang, L. *et al.* Competition between strain and dimensionality effects on the electronic phase transitions in NdNiO_3_ films. *Sci. Rep.* **5**, 18707; doi: 10.1038/srep18707 (2015).

## Supplementary Material

Supplementary Information
