# Supplementary material for: Competition between strain and dimensionality effects on the electronic phase transitions in NdNiO3 films
Source: Sci Rep. 2015 Dec 21;5:18707. doi: 10.1038/srep18707 (PMC4685315; doi:10.1038/srep18707)
Supplement: Supplementary Information [file srep18707-s1.pdf]

Supplementary information for

Competition between strain and dimensionality effects on the electronic phase transitions in $NdNiO_3$ films

Le Wang[1], Sheng Ju[2], Lu You[1], Yajun Qi[3], Yu-wei Guo[1], Peng Ren[1], Yang Zhou[1], & Junling Wang[1]*

[1]*School of Materials Science and Engineering, Nanyang Technological University, Singapore 639798, Singapore.*

[2]*School of Physical Science and Technology, Soochow University, Suzhou 215006, China.*

[3]*School of Materials Science and Engineering, Hubei University, Wuhan 430062, China.*

*Correspondence and requests for materials should be addressed to J.W. (email: jlwang@ntu.edu.sg).

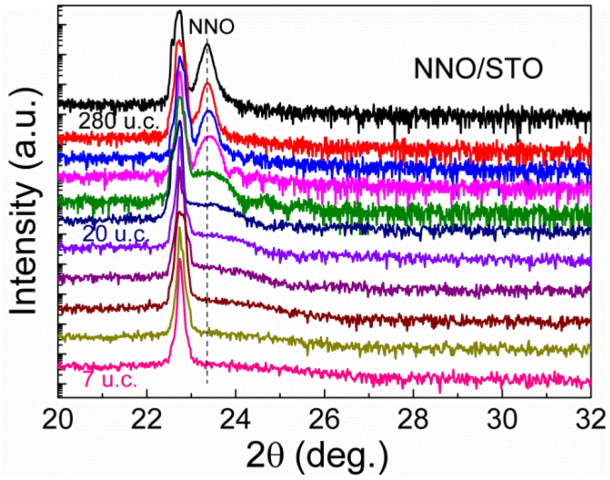

Supplementary Figure S1 | X-ray diffraction patterns of the NNO films grown on STO. As the film thickness decreases, the (001) diffraction peak shifts towards higher 2θ value, indicating the decrease of the out-of-plane lattice constant. The presence of satellite peaks confirms the high quality epitaxial growth. When the film thickness is below 20 u.c., the diffraction peak broadens, leading to increased error bar in the calculated out-of-plane lattice constant.

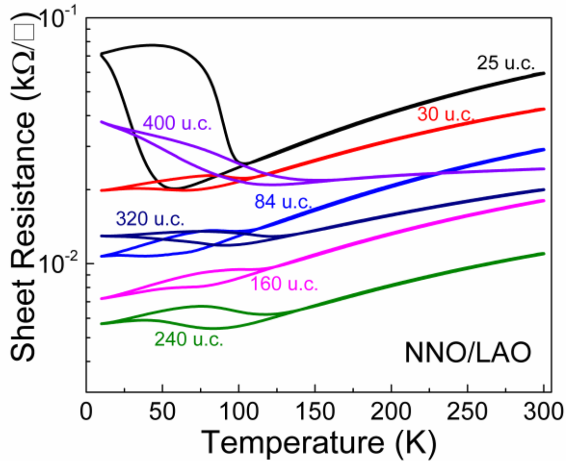

Supplementary Figure S2 | Unusual behavior of NNO/LAO heterostructures with intermediate NNO film thickness (Two-phase region?). For NNO films on LAO, when the thickness is between 25 u.c. and 320 u.c., an unusual behavior is observed. The metal-insulator transition with clear hysteresis can still be observed. However, the low temperature phase appears to be "metallic-like" as shown in the R-T curves (Fig. 3e and Supplementary Fig. S2). Upon reducing temperature, an increase in the sheet resistance at T_{MI} is firstly observed due to the formation of low temperature insulating phase. However, as temperature continues to drop, the sheet resistance decreases even below T_{MI}.

Such unusual behavior has been observed in bulk $PrNiO_3$ under hydrostatic pressure[1,2] and in La-doped NNO[3], and it is denoted the non-Fermi liquid (NFL) state[4,5]. Granados *et al.*[6] proposed that both metallic and insulating phases coexist within a wide temperature range in $PrNiO_3$. Following this model, we suggest that the initial increase in sheet resistance at T_{MI} in our system is due to the formation of the low-temperature insulating phase. However, both metallic and insulating phases coexist at low temperature, and the temperature dependence of the metallic phase dominates as temperature changes[7]. Further experiments, such as low temperature

conductive atomic force microscopic studies, are needed to better understand this unusual behavior. This is beyond the scope of this work.

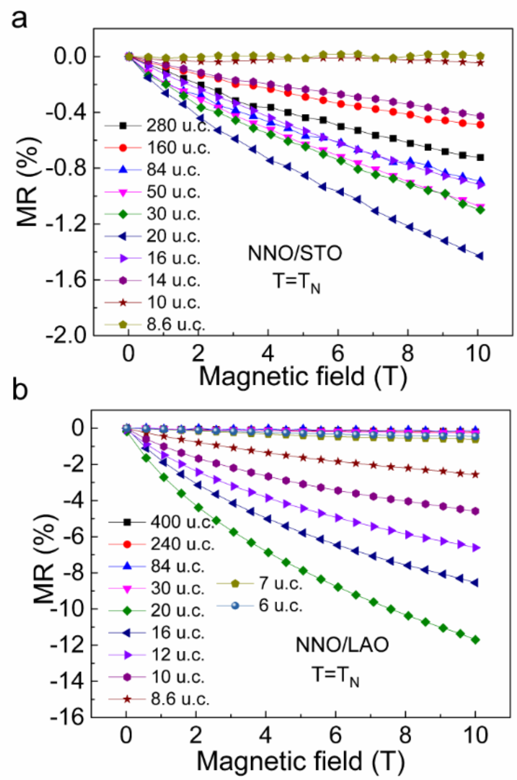

Supplementary Figure S3 | Magnetoresistance (MR) behavior of NNO/STO and NNO/LAO. (**a**) and (**b**) MR as a function of magnetic field perpendicular to the film plane, measured at T_N for the NNO/STO and NNO/LAO heterostructures with different NNO film thickness, respectively. The negative sign of the MR suggests that the low-temperature sheet resistance is not dominated by electron-electron interaction, as that would lead to a positive MR[8,9]. Steve Johnston *et al.* suggest that O *2p* holes provide the metallic charge carriers in nickelates, and strong electron-lattice coupling results in an insulating state[10]. The strong electron-lattice interactions may also be responsible for the negative MR observed in our system.

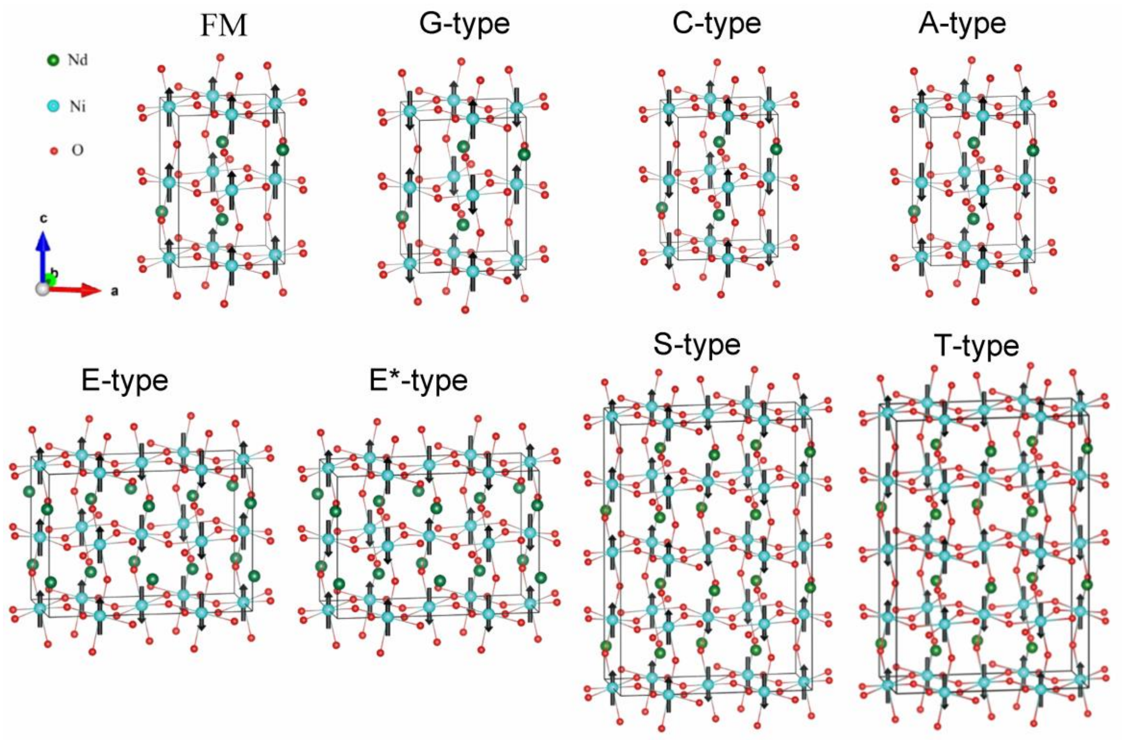

Supplementary Figure S4 | Schematics illustrating the various types of spin arrangements in NNO tested using first principles calculations. Ferromagnetic (FM) ordering, G-type anti-ferromagnetic (AFM) ordering, C-type AFM ordering, A-type AFM ordering, E-type AFM ordering, E*-type AFM ordering, S-type AFM ordering, and T-type AFM ordering, respectively.

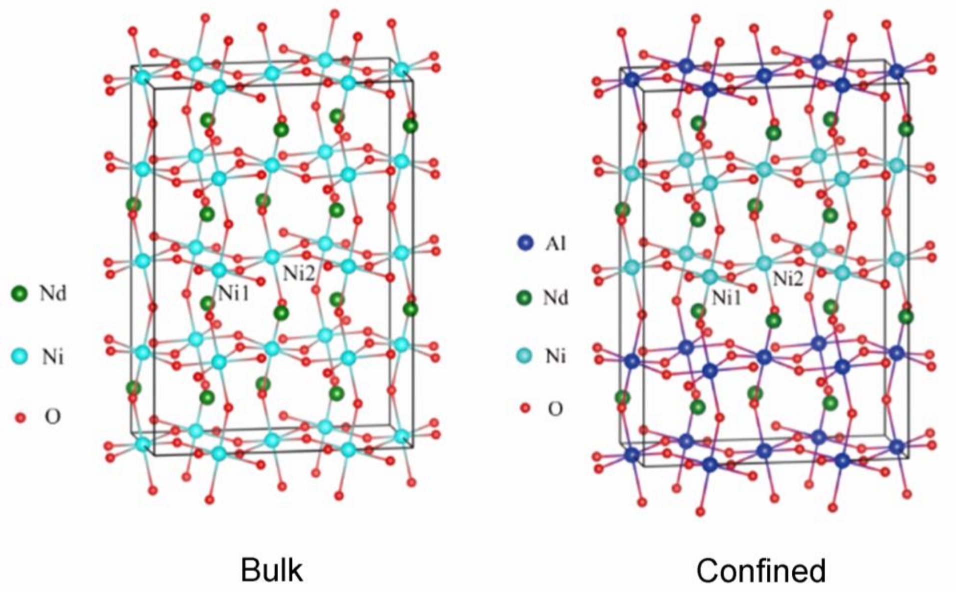

Supplementary Figure S5 | Supercells used in the first principles calculations for bulk NNO (left) and 2D confined NNO (right). We use bulk-strain approach in the calculations. So the crystal structure for NNO films on STO and LAO is the same as bulk, except that the in-plane lattice constants are fixed to that of STO and LAO, respectively.

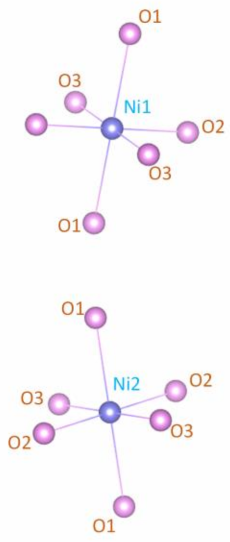

$NdNiO_3$	Bulk	Confined	$LaAlO_3$	$SrTiO_3$
Ni1-O1	1.94427	1.95626/1.92676	1.96303	1.93061
Ni1-O2	1.94851	1.94234/1.94239	1.92851	1.97692
Ni1-O3	1.95709	1.93634/1.93769	1.93392	1.98235
Ni2-O1	1.97344	1.97762/1.98629	1.99137	1.95510
Ni2-O2	1.98708	1.96455/1.95991	1.95673	2.01754
Ni2-O3	1.97885	1.96276/1.97180	1.95124	2.01318
Ni1(m_B)	0	0.116	0.006	0.001
Ni2(m_B)	0.828	0.846	0.763	0.837
Ni1-O1-Ni2	155.7952	155.5093/155.5066	157.7394	152.5803
Ni1-O2-Ni2	154.9309	156.0217/156.3551	155.0406	155.6152
Ni1-O3-Ni2	154.8861	155.9343/156.5254	155.0414	155.4791

Supplementary Table S1 | Theoretical structural parameters for bulk NNO, epitaxially strained thin films on LAO and STO substrates, and confined system. The left figures show the locations of Ni1, Ni2 and various O sites. The Ni-O bond length and the Ni-O-Ni bong angle are given in the table for each system.

References

1 Obradors, X. *et al.* Pressure dependence of the metal-insulator transition in the charge-transfer oxides $RNiO_3$ (R= Pr, Nd, $Nd_{0.7}La_{0.3}$). *Phys. Rev. B* **47,** 12353 (1993).

2 Zhou, J.-S., Goodenough, J. & Dabrowski, B. Pressure-Induced Non-Fermi-Liquid Behavior of $PrNiO_3$. *Phys. Rev. Lett.* **94,** 226602 (2005).

3 Blasco, J. & Garcia, J. A comparative study of the crystallographic, magnetic and electrical properties of the $Nd_{1-x}La_xNiO3-\delta$ system. *J. Phys. Condens. Matter* **6,** 10759 (1994).

4 Doiron-Leyraud, N. *et al.* Fermi-liquid breakdown in the paramagnetic phase of a pure metal. *Nature* **425,** 595-599 (2003).

5 Kim, K., Harrison, N., Jaime, M., Boebinger, G. & Mydosh, J. Magnetic-Field-Induced Quantum Critical Point and Competing Order Parameters in URu2Si2. *Phys. Rev. Lett.* **91,** 256401 (2003).

6 Granados, X., Fontcuberta, J., Obradors, X. & Torrance, J. Metastable metallic state and hysteresis below the metal-insulator transition in $PrNiO_3$. *Phys. Rev. B* **46,** 15683 (1992).

7 Kumar, Y., Choudhary, R., Sharma, S., Knobel, M. & Kumar, R. Strain dependent stabilization of metallic paramagnetic state in epitaxial $NdNiO_3$ thin films. *Appl. Phys. Lett.* **101,** 132101 (2012).

8 A Altshuler, B. L., Aronov, A. G. & Lee, P. Interaction effects in disordered Fermi systems in two dimensions. *Phys. Rev. Lett.* **44,** 1288 (1980).

9 Scherwitzl, R. *et al.* Metal-insulator transition in ultrathin $LaNiO_3$ films. *Phys. Rev. Lett.* **106,** 246403 (2011).

10 Johnston, S., Mukherjee, A., Elfimov, I., Berciu, M. & Sawatzky, G. A. Charge Disproportionation without Charge Transfer in the Rare-Earth-Element Nickelates as a Possible Mechanism for the Metal-Insulator Transition. *Phys. Rev. Lett.* **112,** 106404 (2014).
